# The readiness of emergency and trauma care in low- and middle-income countries: a cross-sectional descriptive study of 42 public hospitals in Albania

**DOI:** 10.1186/s12245-016-0124-5

**Published:** 2016-10-07

**Authors:** Rifat Latifi, Jayleen K. L. Gunn, John A. Stroster, Edmond Zaimi, Fatos Olldashi, Agron Dogjani, Mihal Kerci, Xheladin Draçini, Julian Kuçani, Zhaneta Shatri, Agim Kociraj, Arian Boci, Ross I. Donaldson

**Affiliations:** 1 Department of Surgery, Westchester Medical Center Health, 100 Woods Road, Valhalla, New York, 10595 USA; 2 International Virtual e-Hospital (IVeH) Foundation, Hope, USA; 3 Department of Epidemiology and Biostatistics, University of Arizona, Tucson, AZ 85721 USA; 4 TUHC “Mother Teresa” Hospital, Tirana, Albania; 5 Department of Neurosurgery, University Hospital of Trauma, Tirana, Albania; 6 University Trauma Hospital, Tirana, Albania; 7 Anesthesiology and Intensive Care, University Hospital of Trauma, Tirana, Albania; 8 Faculty of Medicine & Surgery, University of Tirana, Tirana, Albania; 9 The United States Agency for International Development (USAID)/Albania, Tirana, Albania; 10 Department of Emergency Medicine, Harbor-UCLA Medical Center, David Geffen School of Medicine at UCLA, Los Angeles, CA 90095 USA

**Keywords:** World Health Organization, International Association for Trauma and Surgical Intensive Care, Guidelines for Essential Trauma Care, Low and middle-income countries (LMICs), Emergency medical care, Trauma care

## Abstract

**Background:**

Traumatic injuries have become a substantial but neglected epidemic in low- and middle-income countries (LMICs), but emergency rooms (ERs) in these countries are often staffed with healthcare providers who have minimal emergency training and experience. The aim of this paper was to describe the specialized training, available interventions, and the patient management strategies in the ERs in Albanian public hospitals.

**Methods:**

A cross-sectional descriptive study of 42 ERs in the Republic of Albania between September 5, 2014, and December 29, 2014 was performed. Assessment subcategories included the following: (1) specialized training and/or certifications possessed by healthcare providers, (2) interventions performed in the ER, and (3) patient management strategies.

**Results:**

Across the 42 ERs surveyed, less than half (37.1–42.5 %) of physicians and one third of nurses (7.1–26.0 %) working in the ERs received specialized trauma training. About half (47.9–57.1 %) of the ER physicians and one fifth of the nurses (18.3–22.9 %) possessed basic life support certification. This survey demonstrated some significant differences in the emergency medical care provided between primary, secondary, and tertiary hospitals across Albania (the significance level was set at 0.05). Specifically, these differences involved spinal immobilization (*p* = 0.01), FAST scan (*p* = 0.04), splinting (*p* = 0.01), closed reduction of displaced fractures (*p* = 0.02), and nurses performing cardiopulmonary resuscitation (CPR) (*p* = 0.01). Between 50.0 and 71.4 % of the facilities cited a combined lack of training and supplies as the reason for not offering interventions such as rapid sequence induction, needle thoracotomy, chest tube insertion, and thrombolysis. Mass casualty triage was utilized among 39.1 % primary hospitals, 41.7 % of secondary, and 28.6 % of tertiary.

**Conclusions:**

The emergency services in Albania are currently staffed with inadequately trained personnel, who lack the equipment and protocols to meet the needs of the population.

## Background

Progressive modernization and industrialization in low- and middle-income countries (LMICs) has led to a rapid increase in the use of motorized transport, frequently without associated safety policies and country infrastructure able to keep pace. Traumatic injuries have become a substantial but neglected epidemic in LMICs [1]. From 1990 to 2010, injuries represented 11.2 % of disability-adjusted life years (DALYs), a widely accepted measurement of global disease burden [2].

Emergency medicine is a comparatively new specialty, particularly in LMICs, where there is a relative lack of medical staff specifically trained in emergency and trauma care. Often, there are few opportunities to receive such training in resource-limited settings, and those that do exist are usually cost-prohibitive. Human resource shortages also create a lack of local trainers for professional development courses aimed at the healthcare workforce in LMICs [3]. This results in emergency rooms staffed with rotating, off-service physicians or residents who often have minimal training and experience in emergency medicine [4]. A poor primary care system compounds this issue by increasing the acuity of patients presenting to the emergency room [5]. Thus, patients who need specialized emergent care may therefore experience delays in treatment or potentially inadequate care. A lack of structured emergency and trauma systems in LMICs, combined with an increased number of traumas, are major contributors to morbidity and mortality.

The Republic of Albania is an upper-middle-income country in southeastern Europe with three types of hospitals providing emergency services: primary, secondary, and tertiary [6]. Emergency rooms (ERs) associated with primary level hospitals offer access to few medical specialties, with limited laboratory and pathological services. ERs within secondary-level hospitals offer a wider range of specialties and subspecialties, affiliation with nursing schools, and provide teaching activities for allied health personnel. Tertiary hospitals possess ERs with highly specialized personnel affiliated with the medical school and represent the highest level of medical and surgical care in the country. At the tertiary level, the country has five hospitals, each with one or more of their own specialized ERs providing discipline-specific emergency services. The aim of this paper was to describe the specialized training, available interventions, and the patient management strategies in the ERs of Albanian public hospitals.

## Methods

We conducted a cross-sectional study of hospitals in Albania, between September 5, 2014, and December 29, 2014, using a sample of 42 public hospital ERs and a questionnaire based on World Health Organization Guidelines for Essential Trauma Care (EsTC), modified and agreed upon by the Emergency and Trauma Care Physician Working Group of Albania. The University of Arizona’s Intuitional Review Board (Protocol #1509094379), the Ministry of Health in Albania, and the Chief of Staff or Hospital Director in each hospital approved the study.

### Survey measure

EsTC guidelines encompass 260 items of human (staffing, training, skills) and physical (equipment, supplies) resources that are essential or desirable for trauma and emergency care, depending on the level of the facility [7]. We used selected elements from the EsTC guidelines as criteria for assessing emergency and trauma care within Albanian hospitals. Subcategories included the following: (1) specialized training and/or certifications possessed by healthcare providers, (2) interventions performed in the ER, and (3) patient management strategies.

### Statistical analysis

Using STATA version 14.0 (Stata Corporation, College Station, TX), a descriptive analysis was performed, with categorical variables presented as frequencies and percentages and continuous variables as means, standard deviations, and ranges. Fisher’s exact tests for contingency tables were used to test for significance in proportions as the expected cell counts were less than five; the significance level was set at 0.05.

## Results and discussion

Table 1 shows the specialized training of medical personnel within the ERs of the 42 Albanian public hospitals surveyed. Roughly half of doctors in all settings had Basic Life Support (BLS) training, but only about one fifth of nurses. Approximately half of physicians working in the ERs had Pediatric Advanced Life Support (PALS) training at primary hospitals, yet less than one third were trained at secondary and tertiary facilities. Less than half of physicians working in the ERs had received specialized trauma or advanced cardiovascular life support training, while less than one third of nurses possessed equivalent training.

**Table 1:** Specialized training of Albanian medical professionals by emergency room type

	Emergency room type					
	Primary		Secondary		Tertiary	
	(*n* = 23)		(*n* = 12)		(*n* = 7)	
	Yes (%)	No (%)	Yes (%)	No (%)	Yes (%)	No (%)
Basic Life Support (BLS)						
Doctors	56.7	43.3	47.9	52.1	57.1	42.9
Nurses	18.3	81.7	22.9	77.1	21.4	78.6
Pediatric Advanced Life Support (PALS)						
Doctors	53.0	47.0	30.0	70.0	32.9	67.1
Nurses	13.9	86.1	14.2	85.8	14.3	85.7
Advanced Cardiovascular Life Support (ACLS)						
Doctors	44.3	55.7	40.8	59.2	28.6	71.4
Nurses	7.8	92.2	23.0	77.0	12.9	87.1
Trauma Training Course (BTLS/ATLS/PTLS/ITLS)						
Doctors	38.7	61.3	42.5	57.5	37.1	62.9
Nurses	13.9	86.1	26.0	74.0	7.1	92.9

Table 2 presents the few interventions offered in the ERs that reached statistical significance, demonstrating a difference by facility type. Our survey additionally found that 69.6 % of primary hospitals, 75.0 % of secondary hospitals, and 57.1 % of tertiary hospitals were able to provide basic airway management to their ER patients. Three tertiary hospitals, two secondary hospitals, and two primary hospitals described being able to perform endotracheal intubation in the ER on a regular basis. Rapid sequence induction was offered in only one (4.4 %) of the primary ERs and two secondary and tertiary facilities each. Chest tube insertion and needle thoracotomy were not offered due to a combined lack of training and supplies in 52.2 % of primary, 50.0 % of secondary, and 71.4 % of tertiary ERs.

**Table 2:** Interventions offered by emergency room type

	Emergency room type						
	Primary		Regional		Tertiary		
	(*n* = 23)		(*n* = 12)		(*n* = 7)		
	*n*	%	*n*	%	*n*	%	*p* value^a^
Spinal immobilization (cervical collar)							0.01*
Offered in ER	8	34.8	7	58.3	0	0.0	
Not offered—combined lack of training and equipment/meds	9	39.2	2	16.7	7	100.0	
Not offered—lack of training	3	13.0	3	25.0	0	0.0	
Not offered—lack of equipment/medications	3	13.0	0	0.0	0	0.0	
Focused assessment with sonography for trauma (FAST scan)							0.04*
Offered in ER	2	8.7	4	33.3	3	42.9	
Not offered—combined lack of training and equipment/meds	14	60.9	5	41.7	3	42.9	
Not offered—lack of training	0	0.0	0	0.0	0	0.0	
Not offered—lack of equipment/medications	7	30.4	3	25.0	1	14.2	
Splinting performed							0.01*
Offered in ER	11	47.8	9	75.0	0	0.0	
Not offered—combined lack of training and equipment/meds	6	26.1	1	8.4	6	85.7	
Not offered—lack of training	1	4.4	1	8.3	0	0.0	
Not offered—lack of equipment/medications	5	21.7	1	8.3	1	14.3	
Closed reduction of displaced fractures							0.02*
Offered in ER	9	39.1	8	66.7	0	0.0	
Not offered—combined lack of training and equipment/meds	9	39.1	1	8.3	5	71.4	
Not offered—lack of training	3	13.1	2	16.7	0	0.0	
Not offered—lack of equipment/medications	2	8.7	1	8.3	2	28.6	

*p value <0.05 denoted statistical significance

^a^Significance based on Fisher’s exact test

Intraosseous infusion was not offered in 60.9 % of primary, 41.7 % of secondary, and 14.3 % of tertiary hospitals. Central venous access (CVA) within the ER was routinely performed in 57.1 % of tertiary, 16.7 % of secondary, and 17.4 % of primary hospitals. Deep peritoneal lavage was offered in only one ER for each category of facility. Joint dislocation reduction was offered in less than half of the primary and secondary ERs (47.8 and 41.7 %, respectively), while no tertiary hospitals reported offering this intervention, mostly due to a lack of equipment and/or medications. The use of aspirin for suspected or confirmed myocardial infarction was not offered in more than half of the primary and secondary ERs (52.2 and 58.3 %, respectively), whereas only one tertiary facility did not offer this treatment to patients.

Table 3 outlines organizational patient management strategies by ER category. Over half of primary, two thirds of secondary, and all but one tertiary ER had standard triage techniques in place. Mass casualty triage was utilized in the ERs of 39.1 % primary, 41.7 % secondary, and 28.6 % of tertiary facilities. Nurses routinely performed cardiopulmonary resuscitation (CPR) at least some of the time in all of the tertiary hospitals; this was significantly different compared to the situation in 83.4 % of the secondary hospitals and less than half (47.8 %) of the primary hospitals.

**Table 3:** Patient management by emergency room type

	Emergency room type						
	Primary		Secondary		Tertiary		
	(*n* = 23)		(*n* = 12)		(*n* = 7)		
	*n*	%	*n*	%	*n*	%	*p* value^a^
Critically ill patients resuscitated							0.36
No	4	17.4	2	16.7	3	42.9	
Yes	19	82.6	10	83.3	4	57.1	
Standard hospital triage							0.42
No	10	43.5	4	33.3	1	14.3	
Yes	13	56.5	8	66.7	6	85.7	
Mass casualty triage (START)							0.68
No	14	60.9	7	58.3	5	71.4	
Yes	9	39.1	5	41.7	2	28.6	
Vital signs checked routinely							0.08
No	11	47.8	9	75.0	1	14.3	
Yes	12	52.2	3	25.0	6	85.7	
Nurses routinely performed CPR							0.01*
Never	12	52.2	2	16.7	0	0.0	
Yes, sometimes	7	30.4	8	66.7	2	28.6	
Yes, always	4	17.4	2	16.7	5	71.4	
Nurses use bag valve masks							0.11
Never	7	30.4	3	25.0	4	57.1	
Yes, sometimes	11	47.8	7	58.3	0	0.0	
Yes, always	5	21.7	2	16.7	3	42.9	

*p value <0.05 denoted statistical significance

^a^Significance based on Fisher’s exact test

This assessment described a lack of readily available equipment and supplies among Albanian ERs to provide necessary treatments, as well as a need for improvements in the Albanian emergency medicine (EM) training model. There are various designs for EM systems and their corresponding training models around the world, typically classified as either the Anglo-American or the Franco-German systems. The Anglo-American system features skilled physicians in emergency departments and a utilization of paramedics for prehospital emergency medical services. In contrast, the Franco-German system has a highly developed prehospital emergency physician services, but more divided organization at the hospital level. The Franco-German model tends to triage emergencies to different subspecialty areas, with physicians in the ERs possibly not requiring the same breadth of training as those in the Anglo-American system, as they are not exposed to the same variety of emergent cases. Given that these two systems differ substantially in their approach, it is an important consideration when designing improvements to an EM training model [5].

At first glance, the Albanian emergency medical system appears to be more similar to the Franco-German model, as there are no true paramedics and its five tertiary hospitals each have their own specialized ERs. In fact, the main facility, the Mother Teresa Hospital, has six separate ERs, each focusing on its own specialty: internal medicine, infectious diseases, otorhinolaryngology and maxillofacial, surgery, psychiatry, and pediatrics. However, unlike the typical Franco-German model, Albania does not have highly developed prehospital emergency services. Additionally, less than 10 % of injured patients arrive at tertiary hospitals by ambulance, causing multiple problems with the triaging of patients [8]. The recent trend of offering specialized postgraduate emergency medicine training to physicians, as well as a residency program in emergency medicine in Albania, indicates a potential shift more towards the Anglo-American system. It is therefore imperative that any EM training programs developed for Albania take into account the current and future needs of the nation.

Regardless of the model adopted for emergency medicine in Albania, our survey results reveal a lack of equipment, supplies, and/or training regarding several standard treatments and interventions. For example, only four hospitals out of the total 42 surveyed reported being able to perform a needle thoracotomy to treat pneumothorax, while six hospitals in total offered chest tube insertion in the ER. The ERs of one primary hospital and two secondary facilities, but no tertiary hospitals, offered thrombolysis or coronary angiography. Approximately 60 % of physicians working in ERs lack formalized trauma training, while nearly half of the ER physicians in tertiary hospitals and more than three quarters of the nurses lacked BLS training. In order to ensure an adequate number of trained healthcare workers across the entire nation, strategies should include collaboration across different ministries in Albania, such as health, finance, and education [3].

The Distance CME program associated with the Integrated Telemedicine and e-Health Program in Albania (ITeHP-AL) already offers a wide variety of lectures and training opportunities for healthcare workers across the country [9]. We recommend expansion of this established program to include certification courses in basic, advanced, and pediatric life support, as has previously been performed elsewhere [10]. Additionally, given the widespread lack of trauma training and related organizational management structures, such as mass casualty triage, courses in trauma and eventually emergency preparedness and disaster relief would also be most beneficial.

Less than 10 % of the seriously injured are transported to a hospital via ambulance, likely indicating a gap in prehospital emergency care [8]. While the emergency and trauma systems are being improved in Albania, one step that can be taken is to educate laypersons as first responders, which the World Health Organization has recommended as a critical step in establishing effective emergency medical services in LMICs [11]. Not only could the telemedicine infrastructure deliver medical training to healthcare workers but it could also be a low-cost venue to educate and train the citizens of Albania as well.

There continues to be a great divide in LMICs between clinical research findings and their application in everyday clinical practice, an issue that is most crucial for highly effective interventions that are largely ignored. A survey across ten LMICs found that healthcare practitioners are more likely to change their clinical practice if research has been performed and/or published in their own country, compared to outside the country [12]. It is therefore important that LMICs continue to perform high-quality research, rather than merely trying to adopt established standards from abroad. For example, there is a need for studies in LMICs that compare the trauma treatment within hospitals staffed with Advanced Trauma Life Support (ATLS) training to those without ATLS-trained staff in hospitals [13].

Although we surveyed 42 public hospital ERs in Albania, due to a lack of true randomization, our ability to generalize our results across the entire country remains unclear. Our data are also limited by the fact that survey responses were provided by hospital directors and leadership, which constitutes a biased source of reporting, although surveyors attempted to verify responses with tours of each ER. Finally, we did not obtain a list of qualifications and previous training from the Ministry of Health for each physician currently working in an ER, which would have provided a more complete impression of the readiness of these healthcare professionals.

## Conclusions

This study was the first assessment of emergency and trauma service personnel in Albania using the EsTC guidelines. It provides an objective status of 42 different Albanian public hospital ERs across the country and can serve as a guide for focusing future development resources. The assessment has identified several areas for improvement that are essential to decreasing Albania’s high rate of morbidity and mortality from injury and provided suggestions how these deficiencies could be corrected through the modification of existing services.
